# Beyond the Warburg Effect: N-Myc Contributes to Metabolic Reprogramming in Cancer Cells

**DOI:** 10.3389/fonc.2020.00791

**Published:** 2020-05-27

**Authors:** Go J. Yoshida

**Affiliations:** ^1^Department of Pathology and Oncology, Juntendo University School of Medicine, Tokyo, Japan; ^2^Department of Immunological Diagnosis, Juntendo University School of Medicine, Tokyo, Japan

**Keywords:** acyclic retinoid, amino acid transporter, cancer stem-like cells, fatty acid β-oxidation, glutaminolysis, mitochondria, N-Myc, TCA cycle

## Abstract

Cancer cells generate large amounts of lactate derived from glucose regardless of the available oxygen level. Cancer cells finely control ATP synthesis by modulating the uptake of substrates and the activity of enzymes involved in aerobic glycolysis (Warburg effect), which enables them to adapt to the tumor microenvironment. However, increasing evidence suggests that mitochondrial metabolism, including the tricarboxylic acid (TCA) cycle, oxidative phosphorylation (OXPHOS), and glutaminolysis, is paradoxically activated in MYCN-amplified malignancies. Unlike non-amplified cells, MYCN-amplified cancer cells significantly promote OXPHOS-dependent ATP synthesis. Furthermore, tumor cells are differentially dependent on fatty acid β-oxidation (FAO) according to N-Myc status. Therefore, upregulation of FAO-associated enzymes is positively correlated with both N-Myc expression level and poor clinical outcome. This review explores therapeutic strategies targeting cancer stem-like cells for the treatment of tumors associated with MYCN amplification.

## Introduction

N-Myc contains a C-terminal basic region that can bind to DNA and a basic-helix-loop-helix-leucine zipper domain that is responsible for the physical interaction with its counterpart MAX. Myc/MAX heterodimers bind to the DNA sequence 5′-CACGTG-3′, which is termed the consensus E-box (1, 2). N-Myc represses transglutaminase 2 (TG2) transcription in cooperation with specific protein 1 (SP1) and the subsequent recruitment of histone deacetylase 1 (3); however, N-Myc also directly induces the transcription of a specific subset of ATP-binding cassette (ABC) transporter genes. These examples strongly suggest a double-edged sword role for N-Myc in transcriptional regulation according to cell context and tumor microenvironment (4). The Myc family is essential for normal development of the central nervous system (5), and the expression pattern of Myc changes from N-Myc to c-Myc during differentiation to transit-amplifying progenitors (6).

N-Myc is overexpressed in malignant neoplasms of the nervous system, including neuroblastoma, medulloblastoma, glioblastoma multiforme, retinoblastoma, and astrocytoma, as well as in non-neuronal tumors, including hematologic malignancies, small cell lung cancer, neuroendocrine prostate cancer, rhabdomyosarcoma, and Wilms tumors (7). N-Myc is expressed in self-renewing and quiescent hematopoietic stem cells, and expression changes to c-Myc upon differentiation to transit-amplifying progenitors (8). This finding suggests that N-Myc plays a role in the activation of stem cell-like properties characterized by self-renewal potential. Consistently, enforced expression of N-Myc, but not c-Myc, in murine bone marrow cells causes rapid development of acute myeloid leukemia *in vivo* (9). This is supported by the effect of N-Myc upregulation on driving the formation of a neuroendocrine tumor type that differs from c-Myc-driven prostate cancer in histology and response to androgen receptor (AR) signaling-targeted therapies (10, 11). N-Myc suppresses AR signaling and induces polycomb repressive complex 2 (PRC2) target gene repression irrespective of phosphatase and tensin homolog deleted on chromosome 10 (PTEN) status. N-Myc binds to AR enhancers and forms a complex with AR in a manner dependent on its interaction with enhancer of zeste homolog 2 (EZH2). Furthermore, the catalytic activity of EZH2 promotes N-Myc/AR/EZH2-PRC2 complex formation (10). Thus, N-Myc might play a fundamental role in lineage switching from an epithelial origin to a neuroendocrine prostate carcinoma.

Despite its therapeutic potential, targeting N-Myc directly using small molecules remains challenging. As of this writing, there are very few reports demonstrating the efficacy of any compound targeting the binding between N-Myc and MAX, and effective small molecules capable of interfering with the N-Myc/MAX heterodimer *in vivo* have not been identified (7). Several indirect strategies to target N-Myc-driven malignancy are currently being explored, such as impeding MYCN transcription with inhibitors of bromodomain and extraterminal (BET) proteins such as JQ1; targeting proteins that increase N-Myc stability such as allosteric Aurora-A inhibitors; and exploiting synthetic lethal interactions with agents that deregulate N-Myc such as checkpoint kinase 1 (CHK1) inhibitors (7, 12, 13). CHK1 is involved in DNA repair, which is modulated by c-Myc-induced replicative stress (14). CHK1 transcription is markedly elevated in patients with MYCN-amplified neuroblastomas (13). In addition, the MRN complex is composed of RAD50, meiotic recombination 11 (also referred to as MRE11), and NBS1 (also known as nibrin). MRN plays a critical role in processing, sensing, and repairing the double-strand breaks of DNA (15). Petroni and Giannini reported that N-Myc-dependent proliferation of neuroprogenitor cells is accompanied by DNA replication stress, which is attenuated by the MRN complex, a direct transcriptional target of N-Myc. MRN inhibition via mirin also results in the accumulation of DNA damage response (DDR) markers and replication stress-associated DNA foci in an N-Myc-dependent manner. The functional inactivation of the MRN complex mediated by mirin in N-Myc-expressing neural cells fails to induce CHK1 phosphorylation and S phase arrest, whereas it activates both p53 and ATM to trigger apoptotic cell death (16). CCT244747, which is a highly selective and orally active CHK1 inhibitor, has shown therapeutic effects in an N-Myc-driven transgenic murine model of neuroblastoma (17). Zirath et al. (18) demonstrated that the compound 10058-F4, which is thought to disrupt the interaction between c-MYCN-Myc and MAX, impairs respiratory chain and FAO, resulting in apoptosis. A recent study showed *in vitro* that 10058-F4 is effective against acute promyelocytic leukemia and acute lymphoblastic leukemia with c-Myc overexpression (19).

## Metabolic Reprogramming through the Regulation of Amino Acid Transporters by N-Myc

Amino acid transporters contribute to metabolic reprogramming and maintain cancer stem-like phenotypes. xCT (SLC7A11) takes up cystine in exchange for glutamine, which is used for the synthesis of reduced glutathione (GSH) (20, 21), whereas ASCT2 (solute carrier family 1 member 5; SLC1A5) simultaneously takes up glutamine (22, 23). The heterodimer composed of LAT1 (SLC7A5) and CD98 heavy chain (SLC3A2) is broadly and highly expressed in cancer cells and provides essential amino acids characterized by leucine, thereby activating the mammalian target of rapamycin (mTOR) complex1 (24). Oncogenic c-Myc and hypoxia-induced factor 2α (HIF2α) upregulate LAT1 in a coordinated manner, whereas miR-126 suppresses LAT1 expression (25, 26). The leucine influx mediated by LAT1 is associated with another amino acid antiporter, ASCT2 (27). Pharmacological inhibition of ASCT2 suppresses LAT1-mediated leucine uptake, which leads to mTOR signaling inactivation in malignancy (28). Glutamine contributes to the synthesis of α-ketoglutarate (α-KG) via its conversion to glutamate, thereby promoting the tricarboxylic acid (TCA) cycle and the synthesis of nucleotides required for cellular proliferation (27, 29). CD44 variant (CD44v)-positive cancer stem-like cells (CSCs) express high levels of xCT and ASCT2, which promote GSH synthesis from cysteine and α-KG from glutamine, respectively (30). Because c-Myc regulates amino acid transporters such as ASCT2 (23), c-Myc is likely to induce metabolic reprogramming in CD44v-positive CSCs. Collectively, metabolic reprogramming, which is orchestrated by the increased expression and interplay of amino acid transporters, results in glutamine addiction and protects CSCs from redox stress.

**Figure 1:**
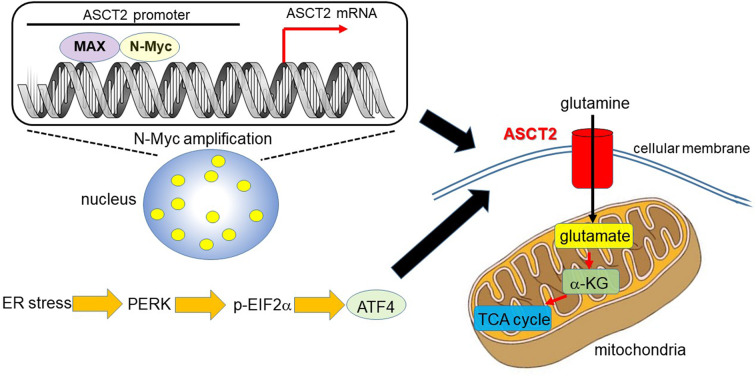
N-Myc and ATF4 act together to upregulate ASCT2, thereby enhancing mitochondrial metabolism. In MYCN-amplified neuroblastoma, the N-Myc/MAX heterodimer binds to the ASCT2 promoter region and aberrantly regulates its transcriptional level (7, 31). Endoplasmic reticulum (ER) stress induces ATF4 via protein kinase-like ER kinase (PERK) and phosphorylated eukaryotic initiation factor 2α (p-EIF2α) (37). ATF4 activates amino acid and glucose metabolism, and promotes protein translation to support increased biosynthetic activities. As such, ATF4 upregulates ASCT2 under ER stress conditions in N-Myc-overexpressing cancer cells. ASCT2 upregulation thus promotes robust uptake of glutamine, which is converted into glutamate and subsequently α-ketoglutarate (α-KG), a substrate of the TCA cycle.

Ren et al. (31) reported that MYCN-amplified neuroblastoma cells prominently depend on ASCT2 to maintain sufficient level of glutamine to activate TCA cycle. MYCN amplification is present in ~30–40% of high-grade neuroblastoma patients and is a poor prognostic factor (32, 33). MYCN-amplified neuroblastoma cells need an efficient machinery to meet the metabolic demands to keep enough amount of glutamine, which is a process which strictly relies on the interaction of specific amino acid transporters. High expression levels of Myc are necessary to maintain the glutaminolytic phenotype and addiction to glutamine as a bioenergetic substrate (23, 34–36). Induction of key regulatory genes encoding glutamine transporters, glutaminase, and lactate dehydrogenase A (LDH-A) is important for glutaminolysis correlated with the Myc-induced increase in glutamine uptake and glutaminase flux. Glutamine addiction is associated with the activity of Myc in redirecting glucose carbon utilization away from mitochondria as a result of LDH-A activation (23, 35). Consequently, Myc-transformed cells become dependent on glutamine anapleurosis to maintain mitochondrial integrity and TCA cycle function. Indeed, MYCN-amplified neuroblastoma cells display addiction to glutamine metabolism (36). Ren et al. (31) identified a well-conserved N-Myc binding site in the ASCT2 promoter region. Although N-Myc and ATF4 cooperatively transactivate ASCT2, this amino acid transporter is significantly upregulated in response to glucose and/or glutamine deprivation and endoplasmic reticulum (ER) stress (Figure 1).

## Mitochondrial Metabolic Reprogramming in N-Myc-Driven Cancer Cells

Alptekin et al. proposed an alternative therapeutic strategy against MYCN-amplified neuroblastoma. These authors demonstrated that upregulation of genes associated with the serine-glycine-one-carbon (SGOC) metabolic pathway underlies the excessive dependence on glycine decarboxylase (GLDC) (38). N-Myc activates GLDC transcription and is essential for maintaining high levels of GLDC expression in MYCN-amplified neuroblastoma cells, suggesting that GLDC and other SGOC pathway genes are cooperatively upregulated (38). Xia et al. reported that the SGOC pathway is inhibited by N-Myc-dependent metabolic vulnerability (39). Genes encoding SGOC-associated enzymes including PHGDH, PSAT1, PHSH, and SHMT are direct transcriptional targets of ATF4. The SGOC pathway provides potential targets for preventing therapeutic resistance in neuroblastoma (40). Increasing evidence supports the pathophysiological significance of GLDC upregulation (41, 42). Increased expression of GLDC in CSCs from non-small-cell lung cancer is essential for their proliferation and tumorigenic ability by driving glycolysis, pyrimidine biosynthesis, and sarcosine production (41). In addition, GLDC upregulation is essential for the viability and growth of glioblastoma cells expressing high levels of serine hydroxymethyltransferase 2, which changes serine into glycine in mitochondria (42). GLDC contributes to metabolic reprogramming exclusively in MYCN-amplified neuroblastoma cells, as demonstrated by the effect of GLDC knockdown on central carbon metabolism pathways, including glycolysis and the TCA cycle, as well as lipid synthesis. GLDC knockout decreases the levels of glucose-6-phosphate, 3-phosphoglycerate, and lactate (38), suggesting that GLDC drives aerobic glycolysis. In addition, GLDC knockdown inhibits the glutamine-dependent reductive carboxylation pathway (38). Glutaminolysis and the reductive carboxylation pathway contribute to the synthesis of acetyl-CoA for lipid synthesis in tumor cells (27, 43, 44). Consistent with this finding, GLDC knockdown considerably decreases levels of fatty acids (palmate and myristic acid) and sterols (lanosterol and cholesterol) (38).

MYCN-amplified neuroblastoma cells exhibit enhanced expression of genes and proteins involved in aerobic glycolysis (Warburg effect), oxidative phosphorylation (OXPHOS), detoxification of reactive oxygen species (ROS), and FAO (45). In MYCN-amplified tumor cells, glycolytic enzymes including hexokinase isoform 2 are upregulated, and enzymes associated with the TCA cycle and the electron transport chain, such as citrate synthase and isocitrate dehydrogenase isoform 2, are expressed at high levels. Increased expression of N-Myc-induced respiratory subunit genes is correlated with adverse clinical outcome in patients with neuroblastoma (45). A moderate increase in ROS in malignant neoplasms modulates cancer cellular signaling via the oxidation of cysteine. H_2_O_2_ inactivates PTEN, a widely-known tumor suppressor, by oxidizing cysteine residues in the active site; this results in the formation of a disulfide bond, which prevents PTEN from inactivating the phosphatidylinositol-3-kinase (PI3K) signaling pathway (46). Therefore, cancer cells finely maintain ROS level within a narrow window which stimulates cellular proliferation. Because most mitochondrial proteins are overexpressed in MYCN-amplified neuroblastoma, and OXPHOS results in the production of intracellular ROS in mitochondria (47), the members of the peroxiredoxin ROS scavenger system, such as peroxiredoxin 6, are upregulated (45). Cancer cells tend to produce large amounts of lactate from glucose, regardless of the available oxygen level, and they activate glycolytic metabolism even before exposure to hypoxic conditions (27, 48). However, neoplastic cells highly depend on the TCA cycle and OXPHOS in mitochondria, rather than on aerobic glycolysis in cytoplasm (27, 49). The metabolic symbiosis between tumor cells and cancer-associated fibroblasts (CAFs) needs the expression of different subtypes of monocarboxylate transporter (MCT) by each cell population. Epithelial tumor cells typically express MCT1, which promotes the uptake of lactate generated and provided by MCT4-expressing and caveolin1-negative CAFs (50, 51). Cancer cells synthesize pyruvate from lactate, providing the TCA cycle and subsequent OXPHOS with an intermediate metabolite. Oliynyk et al. (45) demonstrated that MYCN-amplified neuroblastoma cells depend on both aerobic glycolysis and the TCA cycle; however, metabolic reprogramming driven by N-Myc may rely mostly on mitochondrial metabolism. Furthermore, N-Myc overexpression is related to the FAO process. Indeed, acyl-CoA dehydrogenase is involved in regulating the first step of FAO and is negatively correlated with clinical outcome of patients with MYCN-amplified malignancy (45). Accordingly, the FAO inhibitor etomoxir, which inhibits the rate-limiting FAO enzyme carnitine palmitoyltransferase 1 (CPT1C), suppresses the growth of xenograft tumors derived from MYCN-amplified neuroblastoma (45). This finding suggests that neuroblastoma cells are differentially susceptible to FAO inhibition according to N-Myc expression.

## Lipid Metabolic Reprogramming in N-Myc-Driven Tumor Cells

High expression levels of CPT1C, a brain-specific metabolic enzyme, in N-Myc-positive neuroblastoma cells suggest that increased FAO might be an important metabolic feature in this malignancy (52). While CPT1A and CPT1B exist on the cytosolic surface of mitochondria, CPT1C is localized in endoplasmic reticulum; CPT catalyzes the synthesis of acyl-carnitine from acyl-CoA and promotes lipid conveyance through the membrane of mitochondria (52, 53). The production of acyl-carnitine represents a pivotal regulatory step before FAO because both CPT1A and CPT1C are likely to be inhibited by malonyl-CoA, a final metabolite of the FAO of acyl-CoA molecules. Malonyl-CoA, a fatty acid precursor, simultaneously promotes fatty acid biosynthesis and inhibits fatty acid catabolism, thereby regulating the balance of intracellular fatty acids (18, 54). MYCN-amplified neuroblastoma cells show high levels of CPT1C expression, and inhibition of both CPT1C and peroxisomal β-oxidation leads to lipid accumulation (18). Furthermore, impairment of bromodomain-containing protein 4 (BRD4) due to JQ1 significantly downregulates N-Myc in association with the formation of lipid droplets (18). These findings strongly suggest that N-Myc contributes to lipid metabolic reprogramming, and N-Myc inhibition is responsible for lipid accumulation. Treatment with 10058-F4, which inhibits the c-Myc/MAX complex and the N-Myc/MAX interaction, downregulates several enzymes directly involved in FAO as well as glycolysis and the TCA cycle. High expression levels of FAO-associated enzymes are correlated with robust N-Myc activity and poor clinical outcome in patients with neuroblastoma (18).

Qin et al. (55) reported that signaling networks that regulate ER stress, characterized by the endocannabinoid cancer inhibition pathway, are regulated by stearoyl-CoA desaturase-1 (SCD1) in hepatic tumor-initiating cells with high N-Myc expression. SCD1 is a rate-limiting enzyme which contributes to the synthesis of monounsaturated fatty acids. Deletion of the gene encoding SCD1 increases the rate of FAO mediated by the AMP-activated protein kinase (AMPK) pathway and the upregulation of enzymes necessary for FAO (56). AMPK phosphorylation significantly increases when the function of SCD1 is inhibited, and this inhibition of SCD1 activity has favorable effects for lipid metabolism, such as attenuated lipogenesis and/or increased FAO; these effects are partly attributed to an increase in AMPK activation (57). Changes in AMPK phosphorylation caused by SCD1 up and downregulation affect NAD^+^ levels following changes in NAD^+^-dependent deacetylase sirtuin-1 activity and epigenetic alterations characterized by histone H3 residue 9 acetylation and methylation status (56).

Deregulated N-Myc requires MondoA for lipid metabolic reprogramming in Myc-driven tumors (58). MondoA is associated with the outer membrane of mitochondria, where it can sense both glycolytic intermediate metabolites characterized by glucose 6-phosphate and mitochondrial metabolites necessary for glutaminolysis (59, 60). Metabolites drive the nuclear localization of MondoA, which activates the transcription of genes related to glucose metabolism (61). Depletion of MondoA inhibits N-Myc-induced glutamine uptake, glutaminolysis, and glutamine-derived lipid biosynthesis, which is why apoptosis occurs in the absence of MondoA (58). There is a collaboration network between the nutrient-utilizing N-Myc and the nutrient-sensing MondoA, and the orchestrated interaction between N-Myc and MondoA is critical for amino acid transport, lipid metabolism, nucleotide biosynthesis, and mitochondrial biogenesis. MondoA depletion is responsible for the significant downregulation of fatty acid synthase (FASN), stearoyl-CoA desaturase (SCD), and sterol regulatory element- binding protein-1 (SREBP-1). Metabolite set enrichment analysis indicates that N-Myc activation broadly alters the cellular metabolic characteristics, promotes fundamental changes in amino acid metabolism, and results in an increased amount of precursors for *de novo* lipid and purine biosynthesis (58). *De novo* lipogenesis in N-Myc overexpressing cancer cells depends on MondoA, which is required for N-Myc-induced expression of SREBP-1, FASN, and SCD. The observation that oleate (C18:1) can partially rescue the synthetic lethal effect of N-Myc overexpressing cancer cells lacking N-Myc and MondoA strongly suggests the pivotal role of lipogenesis. Inhibitors of fatty acid synthesis are toxic to N-Myc overexpressing tumor cells (58). Taken together, these findings indicate that metabolic pathways downstream of N-Myc and MondoA, particularly SREBP-1-dependent lypogenesis, are crucial for the survival of N-Myc overexpressing carcinoma.

## The Novel Significance of N-Myc in Hepatic Cancer Stem Cells

**Figure 2:**
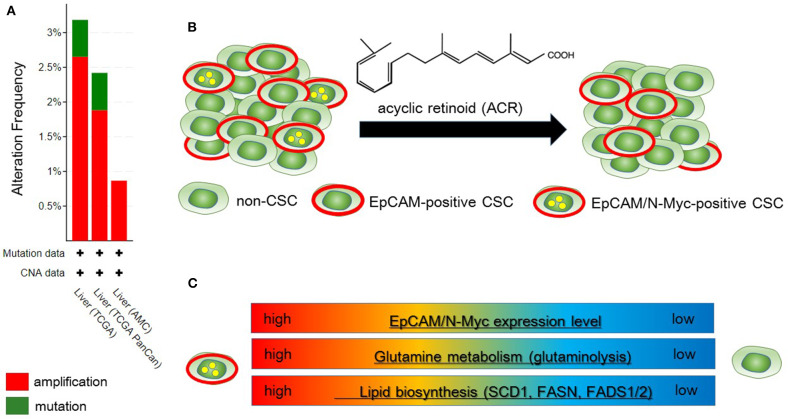
Metabolic reprogramming in N-Myc-positive hepatic cancer stem-like cells (CSCs) is a potential therapeutic target. **(A)** According to the Cancer Genome Atlas data from the cBio Cancer Genomics Portal at Memorial Sloane Kettering Cancer Center (http://www.cbioportal.org/), ~2.5% of hepatocellular carcinoma (HCC) patients have MYCN amplification. **(B)** Primary HCC tissues include both EpCAM-positive and EpCAM/N-Myc double-positive hepatic CSCs (left). Treatment with acyclic retinoid selectively eliminates N-Myc-positive CSCs (right) (64). **(C)** Glutamine metabolism as well as lipid metabolism with SCD1 and fatty acid synthase (FASN) are activated in EpCAM/N-Myc-positive hepatic CSCs (64, 65).

Qin et al. identified N-Myc as one of the hepatic CSC markers in parallel with α-fetoprotein (AFP), epithelial cell adhesion molecule (EpCAM), CD90, CD133, delta-like 1 homolog, and glypican 3 (62–64), and N-Myc plays a pathological role in recurrence of *de novo* hepatocellular carcinoma (HCC). N-Myc is highly expressed in hepatic CSCs compared with non-CSCs. Furthermore, MYCN amplification occurs in at most 2.5% of HCC patients (Figure 2A). In *de novo* HCC, there is a positive correlation between the expression of N-Myc and that of canonical Wnt signaling target molecules such as EpCAM (64, 66), suggesting that N-Myc contributes to the stemness of HCC in cooperation with Wnt/β-catenin signaling. There is no correlation between the expression levels of c-Myc and N-Myc in *de novo* HCC. Alterations of fatty acid metabolism associated with metabolic reprogramming play a pivotal role in facilitating carcinogenesis in the liver (67). HCC cells import fatty acids and other lipids from the bloodstream; however, HCC cells upregulate enzymes involved in the biosynthesis of fatty acids, including SREBP-1-regulated genes including ATP citrate lyase (ACLY), acetyl-CoA carboxylase (ACC), FAS, and SCD-1, leading to the *de novo* synthesis of fatty acids (68). Lipid droplets are considered to be intracellular spherical organelles which are surrounded by a phospholipid single layer (69). The generation of lipid droplets is promoted by hypoxia related to HIF1- and HIF2-mediated repression of CPT1A, an essential enzyme involved in mitochondrial FAO (70). In addition, lipid-modifying enzymes that convert saturated fatty acid (SFA) to monounsaturated fatty acids (MUFA) are responsible for carcinogenesis. This is partly because the ratio of long chain n6-polyunsaturated fatty acids to n3-polyunsaturated fatty acids is associated with a higher risk of HCC development in non-alcoholic steatohepatitis (NASH) model mice (71).

Qin et al. investigated the therapeutic effect of acyclic retinoid (ACR), a synthetic retinoid X receptor α-ligand, against hepatic CSCs expressing high levels of N-Myc. Hepatic CSCs with high expression levels of both EpCAM and N-Myc are more susceptible to ACR than non-CSCs negative for N-Myc expression (64). In general, inhibition of retinoid signaling in the liver is associated with the rapid progression of HCC associated with ROS. Thus, retinoids play crucial roles in mediating lipid metabolism in normal hepatic cells, and altered retinoid-inducing signaling is related to the progression of non-alcoholic fatty liver disease/NASH (NAFLD/NASH) (72). Sp1 may promote the transcription of N-Myc in collaboration with E2F, whereas ACR-induced nuclear translocation of transglutaminase 2 inhibits N-Myc expression in association with the inactivation of Sp1 (64, 73). ACR is a promising vitamin A-like compound for the chemoprevention of HCC because it selectively kills N-Myc-overexpressing CSCs (Figure 2B). ACR has chemopreventive effects on HCC mediated by the inhibition of the hyper-phosphorylation of retinoid receptors and lipid metabolic reprogramming (74, 75). Indeed, lipid metabolic reprogramming is required for the initiation step of HCC tumorigenesis (64, 75). Because lipid biogenesis as well as glutaminolysis are essential for the proliferation of N-Myc-driven cancer cells (Figure 2C), inhibitors of fatty acid synthesis show specific toxicity to malignancy with high expression level of N-Myc (58, 76). The growth-suppressive activity of ACR in HCC cells involves upregulation of pyruvate dehydrogenase kinase 4, which decreases the flux of glycolytic carbon into OXPHOS in mitochondria (75, 77). This response switches the energy source from glucose to fatty acids to maintain the stable ATP production. Although synthetic inhibitors of unsaturated fatty acids (UFAs) such as oleic acid are toxic to N-Myc-overexpressing cells, UFA treatment partially rescues apoptosis induced by knockdown of MondoA, a nutrient-sensing transcription factor (58). Collectively, these findings indicate that disruption of N-Myc-induced lipid metabolic reprogramming may be responsible for the specific toxicity of ACR to hepatic CSCs (64, 65).

## Conclusions and Perspectives

N-Myc enables metabolic reprogramming of cancer cells, which cannot be simply explained by constitutive aerobic glycolysis (Warburg effect). However, MYCN-amplified cells depend on the TCA cycle and OXPHOS as well as lipid metabolism, rather than the Warburg effect. N-Myc upregulates ASCT2, the amino acid transporter contributing to glutamine addiction. MondoA, a nutrient-sensing transcription factor associated with Myc signaling, plays an important role in maintaining N-Myc-induced glutaminolysis and glutamine-derived lipid biosynthesis. ACR, a leading compound of vitamin A, was recently shown to specifically kill EpCAM-positive liver CSCs expressing high levels of N-Myc. ACR holds much promise for preventing *de novo* HCC recurrence. Such CSC population is enriched in enzymes necessary for lipid desaturation including FADS1/2 and SCD1. Considering the complexity of mitochondrial metabolism, further investigation is warranted to design novel therapeutic strategies targeting metabolic reprogramming triggered by N-Myc.

## Author Contributions

GY searched the articles, wrote the manuscript, and submitted the paper to the journal.

## Conflict of Interest

The author declares that the research was conducted in the absence of any commercial or financial relationships that could be construed as a potential conflict of interest.

## Abbreviations

ACR: acyclic retinoid
α-KG: α-ketoglutarate
AMPK: AMP-activated protein kinase
AR: androgen receptor
BET: bromodomain and extraterminal
CD44v: CD44 variant
CHK1: checkpoint kinase 1
CPT1C: carnitine palmitoyltransferase 1
CSCs: cancer stem-like cells
EpCAM: epithelial cell adhesion molecule
ER: endoplasmic reticulum
EZH2: zeste homolog 2
FAO: fatty acid β-oxidation
GLDC: glycine decarboxylase
HCC: hepatocellular carcinoma
LDH-A: lactate dehydrogenase A
MCT: monocarboxylate transporter
mTOR: mammalian target of rapamycin
NASH: non-alcoholic steatohepatitis
OXPHOS: oxidative phosphorylation
PRC2: polycomb repressive complex 2
PTEN: phosphatase and tensin homolog deleted on chromosome 10
ROS: reactive oxygen species
SCD1: stearoyl-CoA desaturase-1
TCA: tricarboxylic acid
UFA: unsaturated fatty acid.
